# A Comprehensive Update on Pompe Disease: From Existing Therapies to Emerging Curative Strategies

**DOI:** 10.3390/ijms27135726

**Published:** 2026-06-25

**Authors:** Rebeca Estevez Barcia, Cristóbal Colón, Álvaro Hermida-Ameijeiras, Laura López-Valverde, Daniel Rodrigues, Cristina Domínguez-González, Jordi Díaz-Manera, Maria L. Couce, José Victor Alvarez

**Affiliations:** 1 IDIS—Health Research Institute of Santiago de Compostela, 15706 Santiago de Compostela, Spain; rebeca.estevez.barcia@sergas.es (R.E.B.); cristobal.colon.mejeras@sergas.es (C.C.); laura.lopez.valverde@sergas.es (L.L.-V.); daniel.caiola.candeias.pontes.rodrigues@sergas.es (D.R.); 2 Metabolic Unit, Clinical University Hospital of Santiago de Compostela, CIBERER, MetabERN, RICORS, 15706 Santiago de Compostela, Spain; 3 Department of Forensic Sciences, Pathology, Gynecology and Obstetrics, Pediatrics, University of Santiago de Compostela, 15706 Santiago de Compostela, Spain; alvaro.hermida.ameijeiras@sergas.es; 4 Neuromuscular Unit, Department of Neurology, Research Institute Imas12, Biomedical Network Research Centre on Rare Diseases (CIBERER), Hospital Universitario 12 de Octubre, Instituto de Salud Carlos III, 28041 Madrid, Spain; cdgonzalez@salud.madrid.org; 5 The John Walton Muscular Dystrophy Research Center, Newcastle University, Newcastle Upon Tyne NHS Trust, Newcastle upon Tyne NE1 3BZ, UK; jordi.diaz-manera@newcastle.ac.uk; 6 Neuromuscular Disorders Laboratory, Insitut de Recerca de l’Hospital de la Santa Creu i Sant Pau, 08041 Barcelona, Spain; 7 Centro de Investigación Biomédica en Red en Enfermedades Raras (CIBERER), 08041 Barcelona, Spain

**Keywords:** acid alpha-glucosidase deficiency, enzyme replacement therapy, infantile-onset Pompe disease, newborn screening, urinary glucose tetrasaccharide

## Abstract

Pompe disease (PD) is a rare, autosomal recessive neuromuscular disorder caused by mutations in the gene encoding acid alpha-glucosidase (*GAA*). The resulting deficiency in GAA, a lysosomal enzyme, leads to the pathological accumulation of glycogen, primarily in cardiac and skeletal muscles. PD presents as a clinical continuum spanning two major phenotypes: infantile-onset Pompe disease (IOPD), the most severe form, typically characterized by onset before 12 months of age, rapid hypertrophic cardiomyopathy, and severe hypotonia; and late-onset Pompe disease (LOPD), which manifests between 12 months of age and adulthood, and is characterized by progressive axial and proximal muscle weakness and respiratory insufficiency. Enzyme replacement therapy (ERT), available since 2006, has improved survival, particularly in IOPD, but is limited by variable efficacy and limited penetration of the blood–brain barrier, necessitating new approaches. In this comprehensive review, we focus on advances in the understanding and management of PD. First, we explore recent diagnostic advances and the characterization of multisystem involvement in PD. Next, we critically discuss the advantages and limitations of current ERT approaches, and advances achieved with next-generation ERT (avalglucosidase alfa, cipaglucosidase alfa + miglustat). Finally, we summarize cutting-edge, potentially curative strategies, including substrate reduction therapy and novel experimental therapies (e.g., gene therapy) that seek to circumvent the limitations of ERT, provide durable effects, and potentially penetrate the central nervous system.

## 1. Introduction

Glycogen storage disease type II (GSDII), also known as Pompe disease (PD, OMIM #232300), is an autosomal recessive disorder marked by a deficiency of the acid alpha-glucosidase (*GAA*) enzyme, which plays a crucial role in breaking down glycogen into glucose within the lysosome [1].

Historically, the prevalence of PD was estimated at approximately 1 in 40,000 births. Recently implemented newborn screening (NBS) programs have revealed a much higher incidence worldwide, with reported frequencies varying significantly between populations, ranging from 1.0 per 4447 [2] to 1.0 per 37,094 [3] screened individuals. A meta-analysis estimates the global birth prevalence at 1.0 cases per 18,432 live births [4], while a study of 11.6 million newborns estimated a prevalence of 1 in 18,711, with no significant differences observed among European, Hispanic American, and Asian populations [5].

*GAA* (OMIM *606800), located on chromosome 17q25, is the sole gene linked to PD in the Online Mendelian Inheritance in Man database. It encodes a 110 kDa precursor polypeptide that undergoes multiple post-translational modifications in the rough endoplasmic reticulum (ER). Once N-glycosylated and properly folded in the ER, the enzyme is transported to the Golgi apparatus, where it acquires mannose 6-phosphate (M6P), a modification essential for its delivery to lysosomes via the cation-independent mannose 6-phosphate receptor (CI-M6P) pathway. CI-M6P receptors are transmembrane glycoproteins predominantly found in the trans-Golgi network and endosomal compartments, with smaller amounts present on the cell surface. Their ability to cycle through the plasma membrane is particularly important for enzyme replacement therapy (ERT), as it enables the entry of therapeutic enzyme molecules into the cell [6,7].

GAA, enclosed within vesicles that bud from the Golgi apparatus and migrate towards the endosomes, undergoes several proteolytic cleavages and N-glycan modifications that are essential for its maturation and activation. In individuals with PD, the impaired enzyme cannot hydrolyze the α-1,4 and α-1,6 glycosidic bonds of glycogen, resulting in excessive glycogen accumulation inside lysosomes. This glycogen buildup ultimately causes lysosomal dysfunction, swelling, and eventual rupture. However, the pathogenesis of PD extends beyond lysosomal dysfunction. Impaired autophagy, disruptions in lysosome-dependent signaling pathways such as the nutrient-responsive MTORC1 complex, increased oxidative stress, and mitochondrial defects all contribute to the disease process. Together, these factors disrupt normal muscle structure, leading to myofibril displacement [1,8].

In summary, GAA deficiency leads to glycogen accumulation in lysosomes, which in turn causes various cellular dysfunctions and tissue degeneration, especially in cardiac, smooth, and skeletal muscle, resulting in a wide-ranging clinical presentation [9].

## 2. Phenotypes and Phenotypic Heterogeneity

The clinical spectrum of PD is fundamentally determined by the nature of the *GAA* mutations and the resulting levels of residual enzyme activity. This relationship is not strictly linear, particularly in late-onset Pompe disease (LOPD). In these cases, similar levels of residual GAA activity can lead to disparate clinical outcomes, suggesting that disease progression is influenced by additional biological modifiers. These factors give rise to a broad spectrum of clinical presentations that vary in terms of symptom onset and the rate of disease progression [7].

Phenotypic variability in Pompe disease is explained by a complex interplay of factors that extend beyond residual GAA enzymatic activity, with particular emphasis on several secondary cellular abnormalities. Among these, impaired autophagy plays a major role, contributing significantly to muscle atrophy and reduced responsiveness to therapy. In addition, mitochondrial dysfunction, characterized by defective mitophagy and impaired mitochondria capacity to oxidize substrates for ATP production, further exacerbates disease progression [10].

These mechanisms are accompanied by major disturbances in calcium homeostasis, affecting calcium transport, signaling, and binding, as well as by activation of inflammatory pathways and profound metabolic remodeling that alters glycolysis, gluconeogenesis, and lipid metabolism, including sphingolipids and phospholipids.

Because genetic modifiers and epigenetic mechanisms remain largely uncharted territory, the integration of multi-omics data, including genomics, transcriptomics, proteomics, and metabolomics, has emerged as a key strategy to unravel the complex genotype-phenotype relationship and to explain why clinical progression is so unpredictable among patients. Ultimately, this network of systemic dysregulation across key cellular pathways determines the severity of pathological burden and the individual response to therapy [10].

Although part of a continuous disease spectrum, two main phenotypic forms are commonly recognized: a profoundly severe, rapidly fatal infantile-onset form (referred to henceforth as IOPD), for which no cure exists, and a comparatively milder, late-onset form, which can occur with or without cardiac involvement [11].

IOPD typically manifests within the first days or weeks of life and is caused by almost complete GAA deficiency (GAA activity < 1%). It is characterized by marked cardiomegaly and hypertrophic cardiomyopathy, accompanied by skeletal and smooth-muscle damage. This leads to dysphagia and gastrointestinal dysmotility, resulting in marked feeding difficulties, malabsorption, and failure to thrive, as well as generalized hypotonia and early respiratory distress. Progression is rapid, and most affected infants succumb within the first 2 years of life due to combined cardiac and respiratory failure [6,12]. Respiratory decline is driven primarily by weakness of the diaphragm and the abdominal wall muscles, particularly the internal obliques and rectus abdominis. Together, these features clearly distinguish the severe IOPD phenotype from the more variable presentations of LOPD [13].

Within the spectrum of IOPD, cross-reactive immunologic material (CRIM)-negative patients represent a particularly severe phenotype. These individuals carry two deleterious GAA mutations and are unable to produce endogenous enzyme, leading to high antibody titers against treatment and poorer clinical outcomes [1].

The designation “non-classical infantile form” refers to children under 1 year of age who exhibit milder cardiac involvement and less pronounced motor impairment than those with the classical presentation, leading to improved survival prospects and more favorable motor development. LOPD is used to describe cases in which symptoms begin anytime from 12 months of age through adulthood, encompassing childhood-, juvenile-, and adult-onset variants [1]. Accounting for roughly 75–90% of PD cases in clinically diagnosed Western cohorts, LOPD is the less severe form, owing to residual GAA activity (up to approximately 30%) [6]. However, these proportions vary substantially depending on the population and detection method; in East Asian cohorts using newborn screening, the documented prevalence of infantile-onset cases is significantly higher than in clinically ascertained series [14].

The clinical presentation is notably heterogeneous, ranging from minimal muscular symptoms to profound limb-girdle and axial weakness accompanied by severe, chronic respiratory failure. Over time, broader systemic involvement can become evident, with glycogen accumulation documented across multiple tissues. This may account for the observed abnormalities of the vascular (e.g., basilar artery aneurysms and dilation of the ascending aorta) and nervous systems, as well as oro-gastrointestinal, urinary tract, and skeletal alterations and white-matter changes visible on magnetic resonance imaging (MRI) [15]. In practice, the label LOPD is also extended to asymptomatic individuals harboring pathogenic *GAA* variants typically associated with late-onset disease. These individuals may be identified through family screening, elevated serum creatine kinase (CK) levels, or NBS, although their clinical trajectory cannot be reliably predicted at diagnosis [16].

The broad variability in PD expression is closely tied to levels of residual GAA enzyme activity in affected tissues. Certain GAA variants are highly disruptive and result in a complete absence of functional enzyme, whereas others are less damaging and result in varying degrees of residual enzymatic function [17]. Known mutations in the GAA gene are reported in ClinVar (https://www.ncbi.nlm.nih.gov/clinvar (accessed on 21 June 2026)) and the Pompe Variant Database (https://www.pompevariantdatabase.clmz.nl/pompe_mutations_list.php?orderby=aMut_ID1 (accessed on 21 June 2026)). Data from the Pompe Registry indicate that the frequency of GAA mutations varies across regions. Despite comparable birth prevalence among different populations [5], substantial regional differences in GAA variant distribution lead to distinct phenotypic profiles and may influence the proportion of IOPD and LOPD cases identified through newborn screening.

For instance, the c.2560C>T variant is the most common worldwide and in North and Latin America, and relatively common in Europe, but rare in Asia-Pacific and the Middle East. The c.1935C>A variant is the most common in Asia-Pacific and the second most common worldwide, but rare elsewhere. The c.-32-13T>G variant is common globally, especially in Europe, North America, and Latin America, but uncommon in Asia-Pacific and the Middle East [18].

Genetics alone cannot fully explain the phenotypic variability in PD, including the marked intrafamilial clinical diversity, characterized by differences in age at onset, severity, and symptom distribution among individuals with the same variants. Further studies are needed to determine the potential roles of epigenetic and trans-acting factors that may influence lysosomal or skeletal muscle function [19].

## 3. Clinical Assessment and Diagnostic Tools

### 3.1. Biochemical Markers

Biochemical markers, particularly serum CK activity, are generally elevated in patients with PD, although normal CK levels in LOPD do not preclude diagnosis [1]. Additional laboratory abnormalities frequently include elevated levels of aspartate aminotransferase (AST), alanine aminotransferase (ALT) [20], lactate dehydrogenase (LDH) [21], and plasma cardiac troponin T (cTnT) [22]. Urinary glucose tetrasaccharide (Glc4), a glycogen-derived tetrasaccharide, has emerged as a highly specific and informative biomarker for disease burden, progression, and therapeutic response of IOPD, as it reflects the extent of glycogen accumulation in skeletal muscle [21].

Evidence supporting the clinical relevance of Glc4 comes from two pivotal clinical trials (phase I/II and phase II/III) [23,24]. The phase I/II study showed that urinary and plasma Glc4 levels were strongly correlated with motor response to ERT over 52 weeks in infants. Urinary Glc4 levels were markedly elevated prior to treatment and decreased rapidly following ERT initiation, reaching normal or subnormal levels within 2 months and remaining low through the following year. These changes closely paralleled clinical improvement, underscoring the utility of urinary Glc4 as a marker of therapeutic response [23]. The phase II/III trial, conducted in a larger cohort of IOPD patients who were followed for up to 3 years, further validated these findings [24].

Several studies have investigated the most effective methods for measuring Glc4 and its utility as a biomarker in PD:

- Sluiter et al. developed a rapid ultraperformance liquid chromatography-tandem mass spectrometry (UPLC-MS/MS) method for the direct separation of underivatized Glc4 from its M4 isomer. This approach showed a diagnostic specificity of 92%, supporting the utility of urinary Glc4 as a reliable biomarker for early PD diagnosis and monitoring of ERT response. The study included urine samples from 66 GSDII patients (infantile and late-onset), as well as individuals with GSDIII, GSDIV, and GSDIX [25].
- Young et al. confirmed high sensitivity (94%) and specificity (84%) of urinary Glc4 for diagnosis of PD in pediatric and adult patients. They recommend combining Glc4 measurements with GAA enzyme activity quantification for accurate diagnosis. Glc4 also served as a marker for monitoring ERT efficacy, with normalization of Glc4 levels in the first weeks considered a positive prognostic indicator. Complementary imaging techniques such as magnetic resonance spectroscopy can further assess tissue involvement and disease severity [26].
- Bobillo Lobato et al. analyzed Glc4 in urine from 35 PD patients (infantile and adult forms) versus 40 controls using high-performance liquid chromatography (HPLC), normalizing values to creatinine levels. Glc4 levels decreased with age in both patients and controls. ROC (receiver operating characteristic) analysis indicated high diagnostic performance: for infantile PD, a cut-off of 4.925 mmol/mol creatinine yielded 85.7% sensitivity and 85.7% specificity; for adult PD, a cut-off of 1.025 mmol/mol creatinine provided 89.3% sensitivity and 88.5% specificity. Elevated Glc4 was particularly notable in infantile patients [27].
- Ren et al. developed and validated a rapid UPLC-MS/MS (ultra-performance liquid chromatography-tandem mass spectrometry) method for the simultaneous quantification of three urinary saccharide metabolites (1,5-anhydroglucitol, Glc4, and maltotetraose) as biomarkers in patients with glycogen storage diseases type Ib and II [28].
- Domínguez-González et al. evaluated urinary Glc4 as a biomarker of disease progression in LOPD. Glc4 levels were quantified in urine samples by LC-MS/MS and normalized to creatinine levels. In a cohort of 35 patients followed longitudinally, higher baseline Glc4 levels were associated with greater functional decline and muscle fat infiltration, supporting its potential value as a prognostic biomarker [29]. Based on previous studies, Glc4 is useful for both a diagnosis and prognostic biomarker in Pompe disease.

### 3.2. Emerging Molecular Biomarkers

Transcriptomic studies have highlighted muscle-related biomarkers of PD. In IOPD, upregulation of genes linked to regenerating fibers (MYOG), inflammation (TGFβ, TNFα, CD79A), and apoptosis correlates with disease progression and response to ERT [30]. Studies published in 2019 have highlighted the relevance of circulating microRNAs as promising biomarkers [31,32]. Carrasco-Rozas et al. (2019) demonstrated that serum levels of muscle-specific microRNAs, particularly the dystromiRs miR-1-3p, miR-133a-3p, and miR-206a, are significantly increased in IOPD and correlate with muscle involvement and functional measures, supporting their potential role in disease monitoring and follow-up [31]. In parallel, Tarallo et al. (2019) identified a distinct signature of differentially expressed circulating microRNAs and showed that miR-133a levels decrease in response to ERT, suggesting its utility as an adjunct biomarker to assess therapeutic response [32]. Collectively, these studies reinforce the role of circulating microRNAs, providing valuable pathophysiological insight that could potentially complement clinical and functional outcome measures in the longitudinal management of PD. In their 2025 study seeking to identify microRNA biomarkers for early diagnosis of IOPD, Bayrak and Tosun highlighted the hsa-miR-548c-3p microRNA as a potential candidate due to its high number of predicted gene interactions and its strong association with the disease according to consensus algorithms. Notably, hsa-miR-548c-3p has been predicted to bind directly to the 3′-UTR of the GAA gene and has been linked to cardiac cell proliferation, consistent with the hypertrophic cardiomyopathy observed in affected infants [33]. MicroRNAs in Pompe require much more extensive investigation; however, they could potentially serve as prognostic biomarkers for disease progression and clinical outcomes.

### 3.3. A Diagnostic Algorithm

1. Positive NBS or clinical suspicion (hypotonia/cardiomyopathy in infants; limb-girdle or axial weakness and respiratory involvement in LOPD).
2. Initial biochemical evaluation: CK, AST/ALT, LDH, urinary Glc4, and cardiac/respiratory assessment according to phenotype.
3. Confirm reduced GAA enzymatic activity in DBS or leukocytes; consider a second tissue if results and genotype are discordant.
4. Perform GAA molecular testing to confirm biallelic pathogenic/likely pathogenic variants, identify pseudodeficiency alleles and guide counseling.
5. Establish baseline disease burden with ECG/echocardiography, respiratory testing, functional scales and muscle MRI as clinically appropriate.
6. In presymptomatic NBS-detected LOPD, use longitudinal monitoring rather than automatic ERT initiation unless objective evidence of disease activity appears.

### 3.4. Clinical and Functional Assessment

The clinical management of PD requires a comprehensive multidisciplinary approach, as the phenotypic spectrum is often characterized by overlapping systemic involvement. Although marked cardiomegaly and hypertrophic cardiomyopathy (characterized by shortened PR intervals, high-voltage QRS complexes, and increased QT dispersion) are hallmarks of IOPD, baseline cardiac evaluation is also indicated in LOPD patients to identify potential subclinical compromise. Similarly, respiratory monitoring is required across all phenotypes. In LOPD, respiratory involvement is assessed through pulmonary function testing, including measurements of maximum inspiratory pressure, maximum expiratory pressure, and forced vital capacity (FVC). FVC is evaluated in both upright and supine positions, with the latter providing insight into the degree of diaphragmatic weakness [34].

As skeletal muscle impairment is the primary driver of morbidity, objective functional motor assessments are essential for longitudinal follow-up. Standardized tools, such as the 6-Minute Walk Test (6MWT), the Gait, Stairs, Gowers, and Chair (GSGC) scale, and the Quick Motor Function Test (QMFT), provide reproducible measures of muscle endurance and functional capacity [35]. These assessments complement muscle imaging, particularly MRI, which has become a cornerstone for both diagnosis and disease monitoring.

MRI effectively delineates the characteristic pattern of fibro-fatty replacement, typically involving the paraspinal and limb-girdle muscles while initially sparing the gracilis and sartorius muscles. Furthermore, the degree of adipose tissue infiltration, quantifiable through Dixon sequences or T1-weighting, correlates closely with clinical functional loss, establishing MRI as a highly sensitive tool for monitoring disease progression and the therapeutic efficacy of ERT [36]. Given the complexity of these evaluations, current clinical practice relies on standardized management algorithms that integrate these functional and respiratory biomarkers [35] with imaging findings [36] to optimize patient outcomes.

### 3.5. Histopathological Assessment

Histopathological examination typically reveals vacuolar myopathy with glycogen accumulation in lysosomal and cytoplasmic spaces. However, in LOPD, these findings can be strikingly heterogeneous: muscle biopsies may show only subtle changes or even appear normal in up to one-third of cases, meaning that the severity of histological damage does not always correlate with clinical disease burden [37,38]. The vacuoles are diastase-sensitive and stain positively with periodic acid-Schiff (PAS) and acid phosphatase, confirming the glycogen nature of the stored material and its lysosomal origin. However, the diagnostic utility of muscle biopsy in adults is limited and has been questioned due to the marked heterogeneity of pathological findings across different muscles and even among fibers within the same muscle [1]. Despite these limitations, muscle biopsy remains a crucial diagnostic tool in cases of clinical doubt or when other tests are inconclusive [38]. Notably, the presence of acid phosphatase-positive lipofuscin inclusions has been proposed as a novel histological marker, particularly in adult patients [1].

### 3.6. Enzymatic and Molecular Diagnosis

Ultimately, definitive diagnosis requires genetic confirmation through the identification of biallelic pathogenic or likely pathogenic variants in the *GAA* gene. While the diagnostic process often begins with an abnormal NBS result and/or clinical features suggestive of the disease, demonstrating reduced GAA enzymatic activity is a critical step. Enzyme activity can be measured in whole blood samples (EDTA), dried blood spots (DBS), cultured skin fibroblasts, or muscle tissue [39]. Notably, enzyme activity assessment in two different tissues is only indicated when genetic testing is inconclusive or exceptionally negative [37]. PD is diagnosed in a proband by the detection of deficient GAA activity in isolated lymphocytes or mixed leukocytes [39]. In classic IOPD, GAA activity is absent or nearly absent (<1%). By contrast, in other clinical phenotypes such as LOPD, enzyme activity is typically markedly reduced (generally <10% of normal), although residual activity up to approximately 30% may be detectable in some cases. Currently, *GAA* gene analysis is routinely performed not only to confirm diagnosis but also to explore genotype–phenotype correlations, identify asymptomatic carriers within families, and provide appropriate genetic counseling [34].

### 3.7. Newborn Screening Programs

The first nationwide NBS program for PD was initiated in Taiwan in 2005 [40]. This program demonstrated that early screening, particularly for IOPD, enables diagnosis within the first month of life, which can significantly improve long-term outcomes through prompt initiation of ERT. However, the initial phase faced a high false-positive rate, in East Asian populations, the pseudodeficiency phenotype is primarily associated with the GAA cis haplotype c.[1726G>A;2065G>A] (p.[G576S;E689K]). This haplotype is relatively prevalent in East Asian populations, with a homozygous frequency of approximately 3% [41]. It reduces in vitro GAA activity to approximately 11–15% of normal levels without causing clinical disease, thereby complicating primary enzyme-activity-based newborn screening [42]. To address this pseudodeficiency hurdle and minimize overdiagnosis, the Taiwanese program adopted a two-tier biochemical algorithm incorporating clinical and laboratory parameters, including hypotonia, elevated creatine kinase (CK ≥ 250 U/L), and increased left ventricular mass index (LVMI ≥ 80 g/m^2^) [14,40]. In 2015, the National Taiwan University Hospital Newborn Screening Center implemented a four-plex tandem mass spectrometry (MS/MS) lysosomal storage disorder multiplex assay [43]. Because reliance solely on primary MS/MS GAA activity yielded an unsatisfactory positive predictive value and a high false-positive risk, a two-tier strategy remained mandatory. This secondary tier, which incorporates the activity ratio together with immediate GAA gene sequencing to filter out benign pseudodeficiencies, increased the overall positive predictive value and effectively lowered the false-positive rate without delaying the detection of IOPD cases. This precise two-tier algorithm is fundamental to mitigating the risks of overdiagnosis and unnecessary medical interventions in healthy individuals carrying pseudodeficiency variants. Beyond IOPD, NBS also identifies individuals with LOPD, raising challenges regarding follow-up strategies, timing of treatment initiation, and psychosocial implications [14]. Nonetheless in 2008, the Advisory Committee on Heritable Disorders in Newborns and Children (ACHDNC) assessed newborn screening (NBS) for Pompe disease and identified substantial evidence gaps regarding screening accuracy, as well as uncertainties surrounding the benefits and potential harms of presymptomatic diagnosis. As a result, Pompe disease was not recommended for inclusion in the Recommended Uniform Screening Panel (RUSP) at that time. The condition was re-evaluated in 2013 after being renominated, and, supported in part by new evidence presented by an external review workgroup, the ACHDNC ultimately recommended its inclusion in the RUSP. Pompe disease was officially added to the panel in March 2015 [34,44]. A study in the United States estimated that screening approximately 4 million newborns annually would identify around 134 cases of Pompe disease, including 40 cases of IOPD, compared with 36 cases that would be clinically diagnosed in the absence of screening [45]. NBS would also detect approximately 94 cases of LOPD, many of which might remain asymptomatic for decades. Furthermore, early identification of IOPD cases through NBS was estimated to prevent 13 deaths and reduce the number of individuals requiring mechanical ventilation to 26 by 36 months of age [44].

The implementation of NBS has led to increased detection of LOPD, mostly in presymptomatic individuals. This shift has highlighted important clinical challenges related to potential overdiagnosis and the risk of overtreatment. Recent studies have shown that a significant proportion of patients identified through screening may remain asymptomatic for years, requiring only long-term clinical follow-up [46]. In this context, there is ongoing debate regarding the optimal timing for initiating ERT, as clear guidelines for presymptomatic patients are lacking and the benefit of very early treatment in LOPD remains uncertain [47]. Consequently, many current recommendations support close monitoring until the appearance of objective signs of disease progression, such as functional impairment, biomarker changes, or imaging abnormalities, before initiating therapy [48].

The following research findings, involving diverse international cohorts and innovative methodologies, highlight major advances in NBS and in our understanding of the pathophysiology of PD:

- Liao et al. compared the effectiveness of tandem mass spectrometry (MS/MS) versus fluorometry in distinguishing affected PD patients from those with pseudodeficiency, a common challenge in Asian populations. The authors demonstrated that the MS/MS-based assay provides a significantly wider analytical range than the fluorometric method. MS/MS successfully discriminated 96% of newborns with pseudodeficiency and 100% of carriers from those with true IOPD or LOPD. This study concluded that adopting MS/MS for NBS may reduce unnecessary clinical referrals and mitigate family anxiety caused by false-positive results [49].
- Sidorina et al. employed an integrated plasma proteomic and lipidomic approach to investigate the complex pathophysiological mechanisms of PD in 12 patients. The multi-omics analysis identified significant changes in expression in 16 proteins, with greatest dysregulation observed in GPLD1, PON1, LDHB, and PKM. This study also revealed abnormal phospholipid metabolism, characterized by significantly reduced levels of six different lysophosphatidylcholines. These findings indicate that PD involves systemic cellular dysfunction affecting inflammatory responses, antioxidant defenses, and calcium homeostasis, highlighting novel biological signatures for potential clinical application [50].
- Gragnaniello et al. conducted a 7-year NBS program in northeast Italy, encompassing 206,741 neonates. Using MS/MS to measure GAA activity, the study identified an overall PD incidence of 1 in 18,795, detecting both IOPD and LOPD phenotypes. Clinical data demonstrated that early initiation of ERT in IOPD patients resulted in favorable outcomes and zero mortality. The researchers highlighted the utility of Glc4 as a critical biochemical biomarker for rapidly differentiating phenotypes and monitoring therapeutic efficacy during follow-up [4].
- Chang et al. used MS/MS to evaluate the birth prevalence of six lysosomal storage disorders (LSDs) in Shanghai via a pilot NBS program involving 20,108 newborns. The investigation revealed a remarkably high prevalence of LSDs with an overall frequency of 1 in 1856 newborns, identifying Krabbe disease specifically as the most common disorder followed by Fabry and PD. Importantly, almost 90% of confirmed cases were LOPD forms, which require careful genetic counseling and long-term clinical management. The findings support the use of MS/MS as a first-tier screening tool combined with biochemical and molecular genetic analysis to assist in parental counseling and management decisions [51].

Overall, these findings demonstrate that MS/MS-based NBS has substantially facilitated earlier and more accurate detection of PD, enabling timely therapeutic intervention and better clinical outcomes. In parallel, large-scale screening programs and multi-omics approaches have furthered our understanding of disease prevalence and systemic pathophysiology, supporting the integration of robust biochemical, molecular, and biomarker-based strategies into routine clinical practice.

### 3.8. Newborn Screening in Europe

The implementation of NBS for PD in Europe has largely been driven by pilot studies and regional initiatives rather than nationwide policies. One of the earliest and most influential programs was established in northeast Italy, where a large-scale pilot study screened over 200,000 newborns using MS/MS to measure GAA activity in DBS [4]. This program demonstrated the feasibility of high-throughput screening for reliable identification of both IOPD and LOPD using MS/MS, and revealed a PD incidence of approximately 1:18,000–1:20,000, consistent with global estimates. Importantly, early initiation of ERT in IOPD patients identified through screening resulted in improved cardiac outcomes, reduced mortality, and better motor development. These findings supported the clinical value of NBS and contributed to broader European interest in NBS for PD. Several European countries have subsequently implemented or piloted PD screening. However, unlike the U.S., Europe lacks a unified screening panel, and decisions are made at national or regional levels. In Spain, Galicia is the only region where an NBS for PD program has been formally implemented since 2024.

In Europe, NBS first-tier testing consists of measuring GAA enzymatic activity in DBS obtained from heel prick samples using MS/MS, fluorometry, or microfluidic methods [52]. Second-tier strategies including measurement of urinary Glc4 and *GAA* gene sequencing are increasingly being used to reduce false positives.

## 4. Treatment Approaches

**Figure 1:**
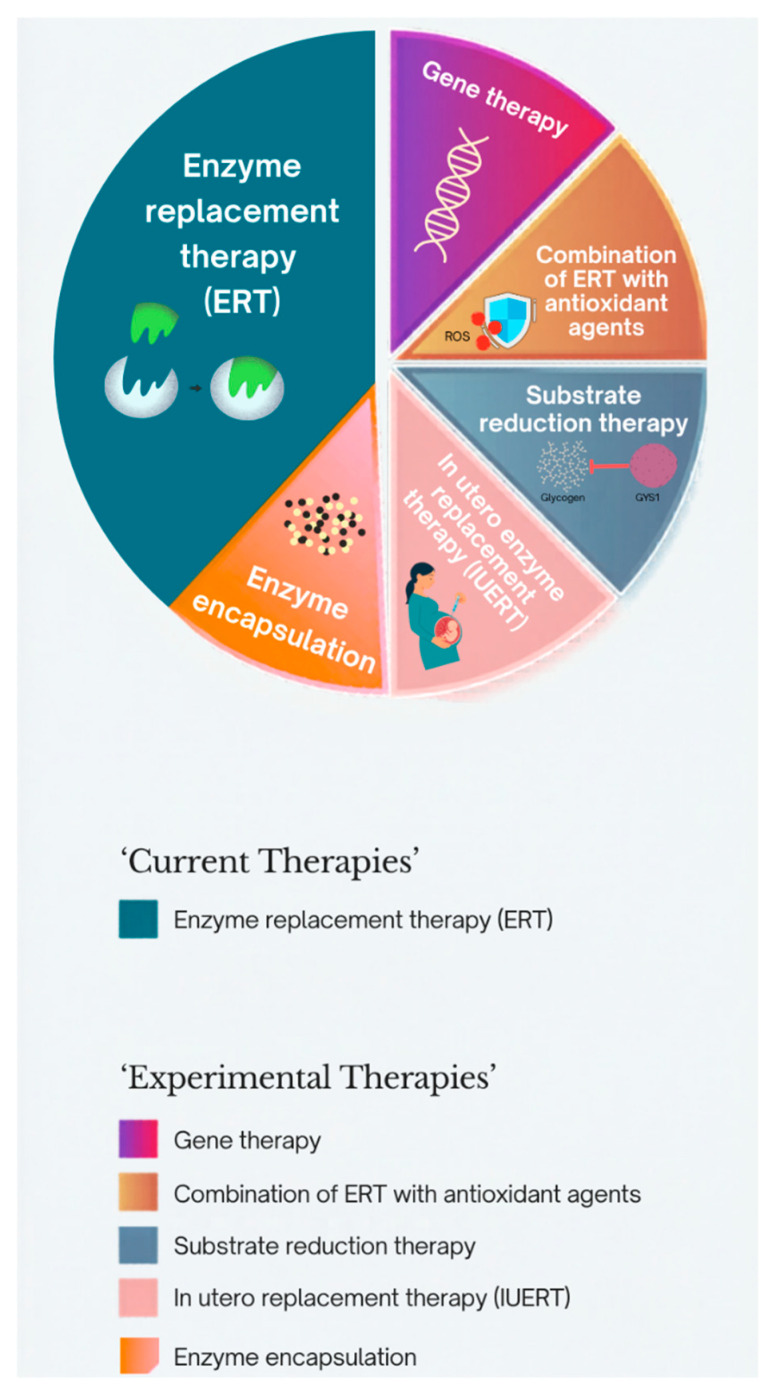
Overview of current and emerging therapeutic strategies for Pompe disease. Schematic depicting approved and investigational therapeutic approaches for Pompe disease, categorized as current standard treatments and emerging experimental strategies.

The therapeutic landscape of PD has expanded considerably in recent years, evolving beyond conventional treatment toward a broader spectrum of innovative strategies aimed at overcoming the limitations of current therapy. Currently approved therapeutic options include ERT, which remains the cornerstone of disease management, and enzyme stabilizer therapy in combination with ERT for selected patients. However, the incomplete efficacy of these approaches, together with limited tissue penetration and the inability of ERT to cross the blood–brain barrier, has prompted the development of several experimental strategies. Emerging investigational approaches include next-generation and prenatal ERT modalities, gene therapy, substrate reduction therapy, and adjunctive antioxidant-based therapies designed to enhance enzyme activity and cellular trafficking. An overview of current and experimental therapeutic strategies for PD is shown in Figure 1.

### 4.1. Enzyme Replacement Therapy (ERT)

In 2006, the therapeutic landscape of PD changed substantially with the approval of ERT consisting of intravenous administration of a recombinant human acid α-glucosidase (rhGAA) precursor, for the treatment of IOPD. Cellular uptake of rhGAA from the circulation is mediated by M6P receptors, which direct the enzyme to lysosomes, where it undergoes proteolytic processing into its active form. In patients with IOPD, treatment with rhGAA markedly improved survival, reduced the need for invasive ventilation, and preserved cardiac function. However, a major therapeutic challenge emerged in CRIM-negative patients because of robust immune responses against rhGAA, which led to the implementation of immunomodulatory regimens to prevent or mitigate this adverse effect [1].

A recent study by Desai et al. showed that NBS facilitated early initiation of ERT (typically within the first 4 weeks of life), which was associated with markedly improved long-term outcomes in IOPD. Favorable outcomes were associated with three main factors: very-early treatment initiation; the use of higher-dose alglucosidase alfa (up to 40 mg/kg/week); and immune tolerance induction (ITI) protocols to reduce the risk of high-sustained antibody titers (HSAT) that neutralize treatment. The choice of immune tolerance induction (ITI) regimen is governed primarily by CRIM status and overall immunological risk. CRIM-negative patients and CRIM-positive patients considered at high risk for sustained anti-rhGAA antibody responses generally receive prophylactic combination ITI consisting of rituximab, methotrexate, and intravenous immunoglobulin (IVIG); bortezomib may be added when clinically indicated. By contrast, transient low-dose methotrexate (TLD-MTX) is used mainly as an adjunctive strategy in standard-risk CRIM-positive patients. With follow-up of up to 18.4 years, 94% of patients achieved independent ambulation, respiratory autonomy, and oral feeding, outcomes biochemically supported by normalization of cardiac mass and a sustained reduction in urinary Glc4 [53].

A recent clinical report described a 3-month-old infant with IOPD who developed generalized urticaria during treatment with alglucosidase alfa. Because therapeutic alternatives were limited, a five-bag, 16-step desensitization protocol lasting 28 h and 35 min was successfully applied, allowing the patient to tolerate the full enzyme dose. This single case report suggests that individualized desensitization may be a feasible strategy for maintaining essential treatment in infants with hypersensitivity reactions to alglucosidase alfa [54].

Optimizing ERT strategies remains essential in PD, given its chronic and progressive course and the fact that, while clinically beneficial, currently available therapies are not curative [55].

Hypersensitivity reactions and incomplete clinical responses observed after alglucosidase alfa treatment underscore the need for improved therapeutic options. While desensitization protocols enable the continuation of life-saving treatment with alglucosidase alfa when no other alternative is available, the development of avalglucosidase alfa represents a significant advancement [1]. Avalglucosidase alfa is generated through the chemical conjugation of an oligosaccharide containing bis-M6P residues to recombinant human GAA using oxime chemistry, thereby enhancing its affinity for CI-M6P receptors and promoting broader distribution across systemic tissues [56]. Following encouraging outcomes from the NEO1 study (in which avalglucosidase alfa demonstrated improved clinical outcomes in both treatment-naïve patients and patients previously treated with long-term alglucosidase alfa) [57], a randomized, double-blind trial was conducted to evaluate the efficacy and safety of avalglucosidase alfa in comparison with alglucosidase alfa in LOPD patients (the COMET trial). In the COMET study, participants received intravenous infusions of either alglucosidase alfa or avalglucosidase alfa at a dose of 20 mg/kg every two weeks. Avalglucosidase alfa met the primary endpoint of non-inferiority in respiratory function, as assessed by upright FVC percent-predicted, with a numerically favorable point estimate compared with alglucosidase alfa, although formal superiority in efficacy was not declared. Additionally, avalglucosidase alfa demonstrated a more favorable safety profile than alglucosidase alfa.

Avalglucosidase alfa was approved by the FDA for the treatment of LOPD in 2021, with a recommended dosage of 20 mg/kg body weight administered biweekly, and of 40 mg/kg for patients weighing less than 30 kg [58].

Notably, a phase 3 clinical trial showed that avalglucosidase alfa is an effective and safe long-term treatment option for LOPD. After nearly 2 years of follow-up, patients treated with avalglucosidase alfa therapy maintained stable respiratory function and physical endurance, and those who switched from their previous treatment also achieved stabilization of motor symptoms and improved quality of life. In addition, avalglucosidase alfa demonstrated a favorable safety profile, with no unexpected risk identified. Taken together, these findings support the use of avalglucosidase alfa to slow the progression of this neuromuscular disease [59].

Long-term data from the COMET trial extension demonstrated that participants treated with avalglucosidase alfa for up to 145 weeks showed clinical improvements in respiratory and motor function, muscle strength, and biochemical markers (Glc4 and serum CK). Notably, patients who switched from alglucosidase alfa to avalglucosidase alfa either improved their clinical status or maintained functional stability, with a favorable safety and immunogenicity profile post-switch. These findings are significant as they support the potential of avalglucosidase alfa to achieve more durable, clinically meaningful outcomes in patients with LOPD [60].

The phase 2 Mini-COMET clinical trial has extended the evidence for safety and efficacy of avalglucosidase alfa in children with IOPD who previously exhibited clinical decline or a suboptimal response to alglucosidase alfa. Over a follow-up period of at least 97 weeks avalglucosidase alfa (20 or 40 mg/kg every other week) was well tolerated, with no deaths or treatment discontinuations due to adverse events. Clinically, all patients achieved stabilization or improvement in motor function and a sustained reduction in biomarkers of disease burden, including CK and urinary Glc4 [61].

In addition to the efficacy observed in previously treated patients, avalglucosidase alfa has shown good results as first-line therapy in infants with atypical IOPD. A clinical case report described how early administration of a 40 mg/kg dose every 2 weeks to a 3-month-old male infant with IOPD, who presented with severe cardiomegaly and respiratory distress, induced a rapid and substantial regression of left ventricular hypertrophy, leading to normalization of cardiac mass after 16 weeks of treatment. This effect is attributed to the mannose-6-phosphate (M6P) content of avalglucosidase alfa, which is approximately 15 times higher than that of alglucosidase alfa, significantly optimizing cellular uptake and lysosomal trafficking of the enzyme in target tissues [62].

These positive cardiac outcomes reported by Uemura et al. [62] are strongly supported by longitudinal evidence indicating that initiating therapy within the first month of life significantly shortens the time required to achieve a normal LVMI and results in superior cardiac remodeling compared to later initiation. Furthermore, data analysis has shown that each month’s delay in starting treatment is associated with an average increase in LVMI of 14.96 units and a 29.6% increase in the odds of developing biventricular hypertrophy, underscoring the vital importance of early detection and immediate therapeutic intervention [63].

Collectively, these findings suggest that this next-generation ERT is not only effective for rescuing patients with LOPD with insufficient responses, but also represents a highly effective primary intervention option to reverse cardiac remodeling and improve overall development in IOPD [62,63].

Recent data suggest that exercise training and nutritional strategies should also be considered to optimize ERT efficacy. In a small cohort of patients with LOPD, introduction of individualized training programs and/or a high-protein diet was associated with improvements in laboratory parameters including CK and LDH levels, as well as increased exercise tolerance [64]. In addition, a randomized controlled study assessing lifestyle interventions in children with IOPD and childhood-onset LOPD (median age, 10 years) demonstrated significant improvements in muscle strength, core stability, muscle function, quality of life, and fatigue after a tailored 12-week intervention program, consisting of physical training combined with a high-protein diet (2 g/kg) in children with PD [65].

In LOPD, studies of patient-derived cells have demonstrated increases in GAA activity, and clinical trials have shown improvements in motor and respiratory function, specifically following treatment with an enzyme stabilizer in combination with ERT (cipaglucosidase alfa plus miglustat, approved in Europe in 2023) [66,67]. This approach is based on the use of a small molecule (miglustat) that binds to and stabilizes the recombinant GAA enzyme, facilitating its correct folding, exit from the endoplasmic reticulum, and arrival at the lysosome, where it can more effectively degrade glycogen. Key advantages of this combination include an oral route of administration, improved enzyme bioavailability, and the stability of the recombinant enzyme, which promote proper intracellular trafficking [67].

The clinical efficacy of this strategy was established in the Phase III PROPEL trial (NCT03729362). Recent post hoc analyses have shown that switching from alglucosidase alfa to cipaglucosidase alfa plus miglustat results in clinically meaningful improvements in motor function (6MWT) and stabilization of respiratory function (FVC). Notably, this dual therapy demonstrated a favorable effect size in patients previously treated with ERT (alglucosidase alfa), suggesting it may overcome the clinical plateau often seen with conventional therapies [68].

3E10 Fab antibody fused to human acid α-glucosidase (FabGAA) represents a novel therapeutic strategy for PD, acting as an antibody-mediated enzyme replacement therapy that can target both lysosomal and cytoplasmic glycogen accumulation. Exploiting the cell-penetrating properties of the 3E10 Fab antibody, this approach offers advantages over conventional rhGAA therapy, which is largely restricted to lysosomal delivery, while displaying comparable efficacy in reducing glycogen levels in preclinical models. This antibody-based platform may therefore improve treatment outcomes by addressing both major glycogen storage compartments involved in disease progression [69].

DNL952 (ETV:GAA) is an investigational, next-generation ERT developed by Denali Therapeutics for the treatment of PD. It consists of a recombinant enzyme (GAA) fused to Denali’s proprietary enzyme transport vehicle (ETV), which is engineered to bind the transferrin receptor (TfR). This mechanism leverages receptor-mediated transcytosis to improve the delivery of the enzyme across the blood–brain barrier and into the muscles, addressing both the neurological and systemic deficits caused by the disease. Denali has initiated a first-in-human Phase I, multicentre, open-label clinical trial (NCT07354724) to evaluate safety, tolerability, pharmacokinetics, and pharmacodynamics of DNL952 in adults with LOPD. All of the above therapies are summarized in Table 1.

### 4.2. Gene Therapy and Other Experimental Therapies

Gene therapy constitutes a potentially definitive treatment, enabling endogenous production of GAA and thereby supporting sustained enzyme availability and natural post-translational modifications for efficient lysosomal trafficking [70]. This approach relies on the mechanism of cross-correction, whereby cells transduced with the vector carrying the *GAA* gene secrete precursor GAA that is subsequently taken up by distant non-transduced cells CI-M6P [6].

Two approaches are described, depending on the type of viral vector used to deliver the *GAA* gene:

i. AAV-Mediated Gene Therapy. Recombinant adeno-associated viral (rAAV) vectors currently represent the most advanced gene therapy platform in clinical development for PD. Systemic administration of AAV vectors in murine models of GAA deficiency (*Gaa* -/-) has demonstrated restoration of GAA enzymatic activity, significant reduction in glycogen levels in heart, diaphragm, tongue and gastrocnemius muscle; and functional improvement of both cardiac and skeletal muscle [71].

Selection of the appropriate gene promoter is a critical determinant of therapeutic efficacy. Muscle-specific promoters (muscle creatine kinase [MCK] or desmin promoters) have been employed to restrict transgene expression to striated muscle and potentially reduce systemic immune response. The investigational gene therapy AT845 (an adeno-associated virus serotype 8 [AAV8] vector containing a codon-optimized GAA transgene under the control of the MCK promoter) demonstrated dose-dependent increases in GAA activity and glycogen clearance in preclinical studies. However, inflammation of the dorsal root ganglia was observed in non-human primates [72]. A clinical trial to evaluate the safety and efficacy of AT845 was launched in 2020. However, following the reported sensory neuronopathy safety signal, further enrollment was halted, and the trial’s current official status is “active, not recruiting” (NCT04174105).

The safety concern highlighted in preclinical studies has also emerged in clinical settings, with sensory neuronopathy reported in a 49-year-old LOPD patient 2 months after receiving an intravenous infusion of AT845. Despite the use of a muscle-specific promoter, the patient developed distal sensory symptoms and gait ataxia, with neurophysiological evidence of pure sensory axonal involvement. These observations indicate that current promoter strategies may not fully eliminate the risk of dorsal root ganglia-related neurotoxicity in humans [73].

An alternative strategy involves liver-directed gene therapy, in which hepatocytes serve as a depot for systemic secretion of GAA. This approach may additionally promote immune tolerance to the transgene product [70]. Clinical programs such as ACTUS-101 [74] are evaluating AAV8-based liver-directed transgenes in patients with LOPD, having demonstrated detectable systemic GAA expression, although these levels may still be insufficient to allow complete replacement of ERT in all treated individuals. In parallel, SPK-3006 has demonstrated robust preclinical efficacy, with sustained systemic presence of GAA and superior glycogen clearance compared with ERT in animal models [75].

To address immune-related challenges, several gene therapy programs incorporate transient immunomodulation strategies aimed at reducing immune responses against the AAV capsid or the transgene product, which remain major barriers to the long-term durability of transgene expression and potential vector re-administration, hence limiting therapeutic efficacy [6]. One study found that administering a non-depleting anti-CD monoclonal antibody successfully suppressed these defensive responses in mouse models. This coreceptor blockade strategy significantly increased GAA enzymatic activity and reduced glycogen buildup in the heart and skeletal muscles. Moreover, the treatment enabled successful re-administration of viral vectors, circumventing cross-immunity between different serotypes. In conclusion, this approach offers a promising avenue for establishing long-lasting immune tolerance [76].

One limitation of AAV-mediated gene therapy for PD concerns the extremely high doses required to systemically target muscle tissue. While muscle growth in pediatric patients poses a known risk of genome dilution, this phenomenon also affects adults due to the ongoing muscle degeneration and subsequent regeneration (driven by satellite cell activation), which leads to loss of the non-integrative episomal transgene. Finally, clinical development of AAV-mediated gene therapy for PD is hindered by potential hepatic toxicity, difficulties in simultaneously correcting all affected tissues (such as the nervous system and skeletal muscle), and a lack of sensitive efficacy measures given the high inter-patient variability [77].

AAV-mediated gene delivery to the central nervous system (CNS) is limited by low transduction efficiency and restricted biodistribution due to the blood–brain barrier. Consequently, effective gene correction in the CNS often relies on indirect mechanisms, such as peripheral transgene expression and protein trafficking, and may still be compromised by immune responses against the transgene [78].

It has been reported that biochemical correction of GAA in animal models of PD after AAV-mediated gene therapy varies depending on the age at treatment. For example, comparisons of gene therapy outcomes in mice with PD treated at 2 weeks versus 2 months of age suggest that adjustment of AAV vector doses may be necessary. Notably, when using similar vector doses, older mice exhibited markedly greater biochemical correction of GAA, along with greater reductions in left ventricular mass and breathing frequency, and increased latency in the wire hang test. These observed functional improvements were more pronounced in females than males, despite a trend toward lower GAA activity correction in females, pointing to potential sex-related differences in therapeutic response [79]. A recent preclinical study reported enhanced GAA activity in female mice with PD following administration of androgen hormone combined with AAV-mediated gene therapy [80]. However, these findings remain limited to animal studies, and the observed benefits appeared modest relative to the systemic adverse effects associated with androgen hormone treatment in females.

ii. Lentivirus-Mediated Gene Therapy (Ex Vivo Hematopoietic Stem and Progenitor Cell (HSPC) Approaches). In PD, trialed ex vivo HSPC-based approaches include hematopoietic stem cell (HSC) lentivirus-mediated gene therapy. This strategy involves isolating patient CD34+ cells from mobilized peripheral blood and transducing them with a lentiviral vector carrying a *GAA* transgene. The modified cells are subsequently reinfused into the patient, who has previously undergone conditioning with an alkylating agent such as busulfan to enable bone marrow repopulation by the modified stem cells capable of secreting functional GAA enzyme [81].

Key advantages of HSC gene therapy include curative potential after a single administration and the induction of immune tolerance. The first clinical trial assessing the efficacy of this strategy was reported in 2009. Results showed partial restoration of GAA enzymatic activity in bone marrow and peripheral blood cells, although reduction in cardiac glycogen accumulation was less effective [82].

The use of a lentiviral (LV) vector containing a stable mammalian promoter together with a human β-globin locus control region resulted in marked improvement of hypertrophic cardiomyopathy in a mouse model of PD, along with a trend toward enhanced motor performance that was not statistically significant when compared with untreated controls at 6 months post-transplantation [83].

In addition to cardiac and skeletal muscle effects, lentivirus-mediated gene therapy in murine models of PD appears to address effects on the CNS. Transplantation of HSCs expressing a codon-optimized GAA transgene led to a reduction in glycogen levels in brain tissue to near normal values. Immunofluorescence analysis of brain sections 3.5 months after transplantation demonstrated GAA expression in a large proportion of microglia and in nearly all astrocytes. This widespread distribution may be related to the capacity of these glial cells to uptake lysosomal enzymes via mannose-6-phosphate (M6P) receptor-mediated pathways, although the exact mechanisms require further clarification [84]. All of the above therapies are summarized in Table 2.

Emerging adjunctive strategies for PD seek to address pathogenic mechanisms beyond GAA deficiency, including increased oxidative stress and impaired autophagy. Given the link between these processes, a recent preclinical study, in tissues from the Pompe disease murine model and in patients’ cells, evaluated the combination of ERT with antioxidant agents. Results suggested that antioxidants may enhance GAA activity, improve enzyme processing, and promote lysosomal trafficking in rhGAA-treated cells [85].

Splice-switching antisense oligonucleotides (AOs) have also been investigated as a targeted therapy for PD, specifically targeting the common c.-32-13T>G mutation, which causes exon 2 skipping in GAA transcripts. Because exon 2 contains the translation start codon, its exclusion leads to mRNA degradation [86]. AOs can modulate splicing by blocking regulatory sequences and promoting exon inclusion. In patient-derived myotubes and fibroblasts, AO-mediated correction restored GAA activity above 20% of normal levels [87]. A subsequent study in myogenic cells derived from 9 patients with LOPD showed AO-dose-dependent increases in GAA activity, although responses varied depending on cell quality and the nature of the second mutation. Clinical validation remains necessary, especially considering that sequence homology in the exon 2 region between humans and mice is only approximately 50% [88].

Substrate reduction therapy has also been proposed as an adjunct to ERT. In particular, antisense-induced exon 6 skipping of the glycogen synthase 1 (GYS1) gene using a phosphorodiamidate morpholino oligonucleotide conjugated to a cell-penetrating peptide (GS-PPMO) significantly reduced skeletal muscle glycogen synthase levels in Pompe mice. These changes resulted in a marked decrease in abnormal lysosomal glycogen accumulation in skeletal, respiratory, and cardiac muscle without causing observable toxicity, supporting substrate reduction therapy as a promising complementary strategy for Pompe disease [89]. In parallel, small-molecule inhibitors of skeletal muscle glycogen synthase, such as substituted pyrazoles, have advanced to Phase I clinical testing in healthy volunteers (NCT05249621) [90]. More recently, a Phase II clinical trial (NCT07123155) has been initiated to evaluate the oral GYS1 inhibitor S-606001 as an add-on to ERT in patients with LOPD, aiming to further reduce glycogen accumulation beyond the capacity of standard ERT.

In utero enzyme replacement therapy (IUERT) has emerged as a promising strategy for early-onset LSDs: advantages of this approach include the ability to start treatment before irreversible organ damage occurs, the potential induction of immune tolerance, and the increased permeability of the blood–brain barrier in utero. The Phase I PEARL trial (NCT04532047), which is currently still recruiting, is evaluating the safety and feasibility of IUERT across several lysosomal disorders with severe early phenotypes [91]. The first clinical evidence of IUERT efficacy in CRIM-negative IOPD was published in 2022. This study described intrauterine administration of alglucosidase alfa followed by standard postnatal therapy, reporting good immunological tolerance, absence of high anti-ERT antibody titers, and favorable clinical evolution, including normal cardiac function and age-appropriate development. Moreover, the authors reported normalization of biochemical biomarkers, specifically a reduction in Glc4 levels in urine samples postnatally, supporting the validity of Glc4 as a biomarker of treatment efficacy [92]. Collectively, these findings reinforce the biological and clinical rationale for prenatal ERT as a potential disease-modifying intervention in severe, early-onset phenotypes.

Given the critical yet often unaddressed alteration of the CNS in IOPD, emerging strategies are focusing on overcoming the limited penetration of current therapies into the CNS. Focused ultrasound has proven a safe means of temporarily opening the blood–brain barrier, allowing the enzyme to reach brain tissue and effectively clear accumulated glycogen [93]. Simultaneously, the discovery of GFAP (glial fibrillary acidic protein) as a blood-based biomarker enables monitoring of neurological damage with greater precision than other indicators, facilitating constant and non-invasive tracking of patient brain health [94]. This combination of a new treatment pathway and an advanced diagnostic tool promises to transform disease management toward a comprehensive approach that truly protects the entire organism.

Nanoparticles for enzyme encapsulation (ERT, Gene Therapy)

One alternative strategy to deliver therapies for PD involves the use of non-viral vectors, most commonly lipid nanoparticles. These vectors offer the possibility of encapsulating enzymes, CRISPR/Cas, RNA, and other therapeutic agents, for internalization into cells, thereby improving their distribution to tissues that free agents cannot reach [95,96]. These nanoparticles are released into endolysosomes, which subsequently fuse with lysosomes [97], facilitating delivery of the enzymes and other systems to their site of therapeutic action. Several articles describe the use of this approach in other lysosomal diseases, including mucopolysaccharidosis [98,99,100], Fabry disease [101], and PD [102]. Furthermore, a variety of routes of administration have been explored, including oral and nasal routes [103].

These strategies, although still experimental, constitute promising therapeutic approaches for this type of disease, addressing the enzyme’s inability to reach target tissues and enabling alternative modes of administration. All of the above therapies are summarized in Table 3.

## 5. Conclusions

ERT remains the mainstay of PD treatment, although additional therapeutic approaches, including combination therapies such as cipaglucosidase alfa plus miglustat, have been approved. Its introduction has profoundly altered the natural history of the disease, significantly improving survival and quality of life. Nonetheless, ERT is not curative, and important challenges persist, including heterogeneous clinical responses and a gradual decline in therapeutic efficacy over time, particularly after the first years of treatment. In addition, limited uptake in specific tissues, particularly skeletal muscle, and the inability of currently available ERTs to cross the blood–brain barrier remain major obstacles to achieving complete disease correction. The development of next-generation ERT formulations may help overcome these limitations by offering improved tissue targeting, enhanced durability, and potentially more sustained clinical benefits.

Gene therapy approaches hold promise for sustained enzyme restoration and potentially lifelong therapeutic benefit following a single administration. While advances in vector design and immunomodulation have overcome several early limitations and have shown the potential to achieve systemic and CNS correction, long-term safety and the durability of clinically meaningful therapeutic effects remain to be fully established.

The implementation of NBS programs enables earlier diagnosis and timely initiation of therapy, potentially before irreversible pathological changes occur. Moreover, these screening efforts have demonstrated that this genetic disorder is more prevalent than previously recognized. However, the increasing identification of late-onset phenotypes and variants of uncertain significance raises important questions regarding prognosis, patient stratification, and the optimal timing of therapeutic intervention. Future advances most likely to transform clinical practice will depend on the development of therapies capable of achieving effective systemic and CNS correction, the integration of precision medicine approaches to optimize treatment selection, and the generation of long-term outcome data to guide individualized management strategies.

## Figures and Tables

**Table 1:** Summary of ERTs for Pompe disease and their key characteristics.

ERT	Description	Key Advantages	Challenges to Overcome
Alglucosidase alfa (1st-Generation ERT)	Intravenous administration of rhGAA precursor	Improves survival and cardiac function in IOPD. Ultra-early use enables long-term ambulation	Clinical plateau effect High immunogenicity risk in CRIM-negative patients
Avalglucosidase alfa (2nd-Generation ERT)	Bis-M6P conjugated rhGAA for enhanced tissue uptake	15-fold higher receptor affinity Rapidly reverses left ventricular hypertrophy in IOPD	Failed to show statistical superiority over 1st-Generation ERT in LOPD (COMET trial)
Cipaglucosidase alfa + Miglustat (Combination)	Intravenous ERT+ oral small-molecule chaperone	Chaperone stabilizes enzyme Overcomes clinical plateau and improves 6MWT	Complex dosing regimen (combined intravenous and oral delivery)
FabGAA (3E10 Fab) (Antibody Fusion)	rhGAA fused to a cell-penetrating antibody fragment	Targets both lysosomal and cytoplasmic glycogen	Restricted to preclinical data; lacks human clinical validation
DNL952 (ETV: GAA) (Transport Vehicle)	rhGAA fused to a transferrin receptor (TfR)-binding vehicle	Crosses the blood–brain barrier Treats systemic and CNS deficits	Early development stage (currently in Phase I trials)

**Table 2:** Summary of gene therapies for Pompe disease and their key characteristics.

Vector /Platform	Candidate /Developer	Promoter/ Target Tissue	Key Advantages	Challenges to Overcome	Trial Identifier	Phase and Status
rAAV8 (Adeno-Associated Virus)	AT845 (zocaglusagene nuzaparvovec) (Astellas Gene Therapies)	Promoter: Muscle-specific (MCK) Target: Striated muscle	High cardiac and skeletal muscle glycogen reduction	Neurotoxicity risk: dorsal root ganglia inflammation observed in non-human primates and sensory neuropathy/gait ataxia in a 49-year-old LOPD patient	NCT04174105	Phase I/II Active, not recruiting (enrollment halted due to safety signal)
AAV2/8- LSPhGAA liver- depot gene therapy	ACTUS-101 (AskBio Inc.)	Promoter: Liver-specific Target: Hepatocytes (systemic depot)	Promotes immune tolerance to the transgene. Demonstrates detectable systemic GAA expression	Requires extremely high doses to target muscle systemically. Current GAA expression levels may still be insufficient to completely replace ERT	NCT03533673	Phase I Completed
rAAV (Adeno-Associated Virus)	SPK-3006 (Spark Therapeutics)	Promoter: Liver-specific Target: Hepatocytes (systemic depot)	Robust preclinical efficacy with sustained systemic GAA presence. Superior glycogen clearance compared with ERT in animal models	Potential hepatic toxicity Risk of episomal transgene genome dilution due to ongoing muscle degeneration/regeneration	NCT04093349	Phase I/II Active, not recruiting (no longer looking for participants)
Lentivirus (Ex Vivo HSPC)	Ex vivo transduction of patient CD34+ cells and reinfusion after conditioning with busulfan	Promoter: Stable mammalian/human β-globin LCR. Target: Bone marrow and peripheral blood	Curative potential after single administration. Immune tolerance. Reduces CNS glycogen to near-normal levels	Requires conditioning with alkylating agents (busulfan). Less effective cardiac glycogen clearance in early clinical tests. Motor improvements lack statistical significance	Early academic trials/Registry dependent	Clinical development

**Table 3:** Summary of other experimental therapies for Pompe disease and their key characteristics.

Other Experimental Therapies	Description	Key Advantages	Challenges to Overcome
Antioxidant Therapy	Combination of ERT with antioxidant agents to address pathogenic mechanisms beyond GAA deficiency (oxidative stress and impaired autophagy)	Enhances GAA enzymatic activity. Improves enzyme processing. Promotes lysosomal trafficking in rhGAA-treated cells.	Preclinical
Splice-switching antisense oligonucleotides (AOs)	Causes exon 2 skipping in GAA transcripts	GAA activity restored above 20%	Clinical validation
Substrate reduction therapy	Antisense-induced exon 6 skipping of the glycogen synthase 1 (GYS1) gene using a phosphorodiamidate morpholino oligonucleotide conjugated to a cell-penetrating peptide (GS-PPMO)	Decrease in abnormal lysosomal glycogen accumulation in skeletal, respiratory, and cardiac muscle No observable toxicity	Early development stage (currently in Phase II trials)
In utero enzyme replacement therapy (IUERT)	Intrauterine administration of rhGAA	Start treatment before irreversible organ damage occurs. Potential induction of immune tolerance. Increased permeability of the blood–brain barrier in utero.	Evaluating the safety and feasibility (Phase I PEARL trial)
Focused ultrasound	Temporarily opening the blood–brain barrier, allowing the enzyme to reach brain tissue and effectively clear accumulated glycogen	Allowing the enzyme to reach brain tissue and effectively clear accumulated glycogen.	Early development stage
Nanoparticles	Encapsulation of enzymes for internalization into cells	Improve the distribution to tissues that free agents cannot reach. Released into endolysosomes, which subsequently fuse with lysosomes, facilitating delivery of the enzymes and other systems to their site of therapeutic action. Possible oral/nasal administration.	Early development stage

## Data Availability

No new data were created or analyzed in this study. Data sharing is not applicable to this article.
